# A Novel High-Potency Tetanus Vaccine

**DOI:** 10.1128/mBio.01668-20

**Published:** 2020-08-11

**Authors:** Amanda Przedpelski, William H. Tepp, Sabine Pellett, Eric A. Johnson, Joseph T. Barbieri

**Affiliations:** a Medical College of Wisconsin, Department of Microbiology and Immunology, Milwaukee, Wisconsin, USA; b University of Wisconsin—Madison, Department of Bacteriology, Madison, Wisconsin, USA; Emory University School of Medicine

**Keywords:** DNA recombination, *Escherichia coli*, immune response, immunization, tetanus toxin

## Abstract

Chemically inactivated tetanus toxoid (CITT) is clinically effective and widely used. However, CITT is a crude nonmalleable vaccine that contains hundreds of Clostridium tetani proteins, and the active component is present in variable and sometimes minor percentages of vaccine mass. Recombinant production of a genetically inactivated tetanus vaccine offers an opportunity to replace and improve the current tetanus vaccine. Previous studies showed the feasibility of engineering full-length tetanus toxin (TT) in Escherichia coli. In the present study, full-length TT was engineered with eight individual amino acid mutations (8MTT) to inactivate catalysis, translocation, and host receptor-binding functions, retaining 99.4% amino acid identity to native tetanus toxin. 8MTT purified as a 150-kDa single-chain protein, which trypsin nicked to a 100-kDa heavy chain and 50-kDa light chain. The 8MTT was not toxic for outbred mice and was >50 million-fold less toxic than native TT. Relative to CITT, 8MTT vaccination elicited a strong immune response and showed good vaccine potency against TT challenge. The strength of the immune response to both vaccines varied among individual outbred mice. These data support 8MTT as a candidate vaccine against tetanus and a malleable candidate conjugate vaccine platform to enhance the immune response to polysaccharides and other macromolecular molecules to facilitate a rapid response to emerging microbial pathogens.

## Abstract (precis)

Chemical inactivation is a clinically effective mechanism to detoxify protein toxins to produce vaccines against microbial infections and to serve as a platform for production of conjugate polysaccharide vaccines. This method is widely used for the production of protein toxin vaccines, including tetanus toxoid. However, chemical modification alters the protein structure with unknown effects on antigenicity. Here, a recombinant full-length tetanus toxin (TT) is engineered with 8 mutations (8MTT) that inactivate three toxin functions: catalysis, translocation, and receptor binding. 8MTT is nontoxic and elicits a potent immune response in outbred mice. 8MTT also represents a malleable platform for the production of conjugate vaccines, which can facilitate a rapid vaccine response against emerging microbial pathogens.

## INTRODUCTION

Vaccination is the most effective means to prevent infectious diseases (1). Chemical inactivation of bacterial toxins has produced several protective vaccines, including diphtheria and tetanus vaccines. These vaccines are based on native diphtheria toxin or tetanus toxin (TT) that are converted to toxoids, diphtheria toxoid or catalytically inactive tetanus toxoid (CITT), respectively, by chemical inactivation with formaldehyde (2). In the United States, the CITT vaccine has reduced the mortality of tetanus by 99% (1, 3). However, neonatal tetanus is a significant disease, with globally ∼1 million cases and ∼34,000 deaths per year according to WHO estimates for 2015 (4–6). Despite the significant public health advances due to widespread CITT applications, the CITT vaccine is crude and possesses varied amounts of CITT (7). In addition, CITT vaccination can be clinically reactive, leading to unwanted side effects after injection (8). A recent assessment of botulinum toxin, which is genetically related to tetanus toxin, found that when formalin conditions are too harsh, botulinum toxoid elicits limited protection against toxin challenge (9).

TT is one of the most toxic proteins for humans (10, 11), with a 50% lethal dose (LD_50_) of ∼2.8 × 10^8^ per mg of protein (9). TT is produced by Clostridium tetani as a single-chain toxin that is proteolytically converted to the active di-chain toxin, comprising an N-terminal protease light chain (LC) linked by a disulfide bond to the C-terminal heavy chain (HC). The HC contains a receptor-binding domain (HCC) and LC translocation domain (HCN) (12). TT enters peripheral motor neurons and traffics to the soma and to synapses of central nervous system interneurons, where TT transcytoses and enters inhibitory neurons. The LC is released into the cell cytosol of inhibitory neurons and cleaves vesicle-associated membrane protein 2 (VAMP2), a vesicle soluble NSF attachment protein receptor (SNARE) (13, 14). VAMP2 cleavage in inhibitory neurons blocks neurotransmitter exocytosis, preventing release of inhibitors of neuromuscular synapse function, leading to continued neuromuscular activation and spastic paralysis (15).

CITT is also one of the primary conjugate vaccine carriers for polysaccharide antigens, including Haemophilus influenzae type b capsular antigen and the serogroup polysaccharides (16–19). Polysaccharide antigens require a protein conjugate to effectively elicit a T-cell-dependent immune response (20, 21). To expand the use of conjugate vaccine applications, other platforms are being developed, including the recombinant diphtheria toxin derivative CRM-197 (22–24). Additional malleable vaccine platforms are needed to expand conjugate vaccine use in the United States and globally (25).

Previous attempts to produce recombinant tetanus vaccines include engineered forms of the HCC (26, 27). Recently, our group engineered a genetically inactivated full-length tetanus toxin, TT(R372A, Y375F) [TT(RY)] (28). The crystal structure of TT(RY) provided the first image of full-length TT (29). In this study, safety of the full-length tetanus vaccine was improved by engineering a genetically inactivated full-length TT (8MTT) with mutations in catalytic, translocation, and host receptor-binding domains. In outbred mice, no toxicity was observed at 0.6 mg 8MTT per mouse, indicating 8MTT is >50 million-fold less toxic than native tetanus toxin. A pilot vaccine study in mice indicated 8MTT is a potent vaccine against native TT challenge and elicits a robust immune response.

## RESULTS

### Properties of 8MTT.

**FIG 1:**
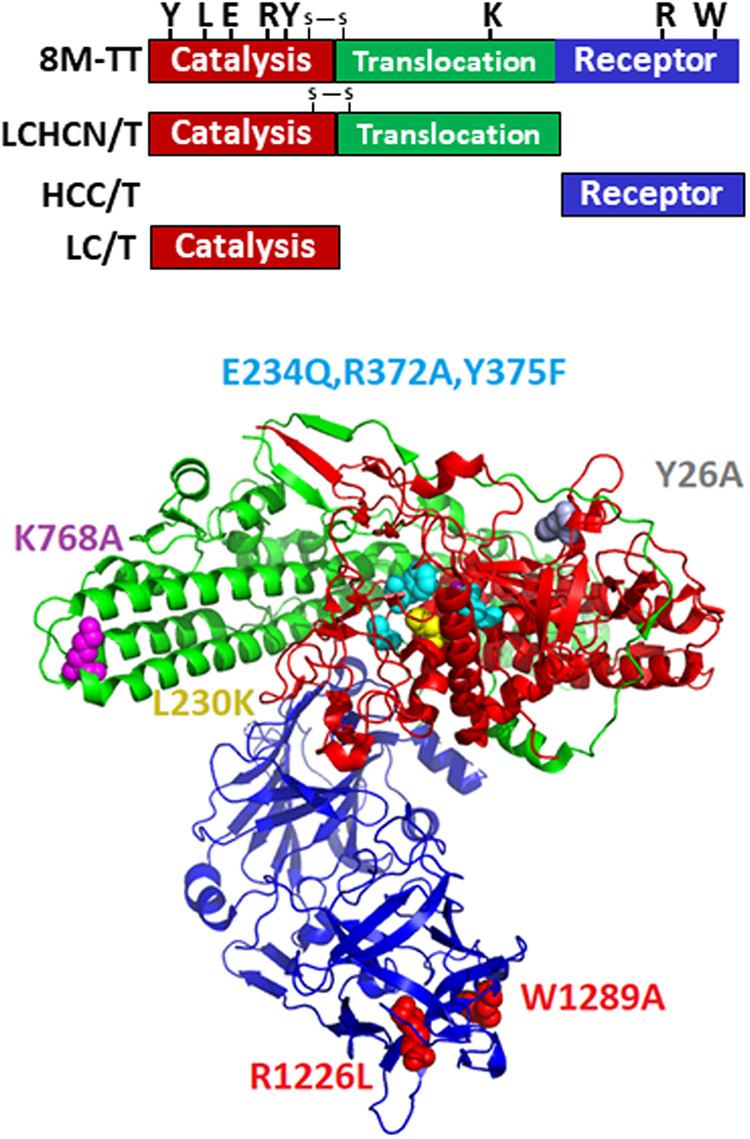
8MTT as a recombinant genetically engineered vaccine. 8MTT was engineered to inactivate the 3 functional components of toxin action: light chain (LC; red, catalysis), substrate-binding Y26A (Y), substrate cleavage L230K (L), zinc binding E234Q (E), R372A (R), and Y375F (Y); heavy chain translocation domain (HCN; green, LC translocation), K768A (K); and heavy chain receptor binding domain (HCC; purple, receptor), dual ganglioside binding R1226L (R) and W1289A (W). (Top) Schematic of TT-derived protein analyzed. (Bottom) 8MTT with eight engineered point mutations within TT(RY) (PDB 5N0B).

**FIG 2:**
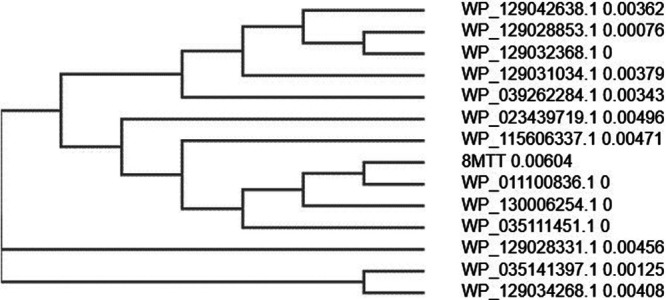
Clustal Omega alignment of 8MTT and native TTs. Phylogenic tree between 8MTT and the known TTs. Input parameters: output guide tree, true; output distance matrix, false; dealign input sequences; false; mBed-like clustering iteration, true; number of iterations, 0; maximum guide tree iterations, −1; maximum hidden Markov model (HMM) iterations, −1; output alignment format, clustal num; output order, aligned; and sequence type, protein.

Previous studies produced a genetically inactivated full-length TT [(TT(RY)] in Escherichia coli (28) that was subsequently crystalized (29). TT(RY) was attenuated, ∼125,000-fold less toxic than native tetanus toxin, which was sufficient to enable biologic and biochemical studies but not for vaccine applications (30). Based on the known action of individual amino acids within TT (Table 1 and Fig. 1), six additional amino acids of TT(RY) were modified to inactivate LC catalysis, LC translocation, and host receptor binding (31–34). The resulting 8MTT differed by 0.6% in primary amino acid sequence with the reference TT sequence (accession number WP_011100836.1) (35), which was the template used to generate 8MTT. The primary amino acid sequence of naturally occurring TT variants differ from 0.3% to 2.6% relative to the reference TT sequence (Table 2). In a Clustal Omega phylogenic alignment (36) (Fig. 2) of the TT variants, 8MTT aligned most closely to the reference TT sequence (accession number WP_011100836.1).

**TABLE 1:** Mutations engineered into 8MTT

Domain	Residue(s)	Function	Reference^*a*^
LC	Y26A	VAMP2 binding	31
LC	L230K	VAMP2 cleavage	31
LC	E234Q, R372A, Y375F	Zinc binding	33
HCN	K768A	LC translocation	34
HCC	R1226L W1289A	Host receptor binding	32

a References describe the basis for using each mutation that neutralize a specific action of tetanus toxin.

**TABLE 2:** RefSeq alignment of TT variants versus TT prototype used as a template to engineer 8MTT^*a*^

Accession no.	Organism on which the RefSeq protein is annotated	Protein length (aa)^*b*^	% sequence identity between model protein and candidate protein	No. of RefSeq assemblies on protein annotated
WP_011100836.1	Clostridium tetani	1,315	100	13
WP_023439719.1	Clostridium tetani	1,315	98.78	1
WP_035111451.1	Clostridium tetani	1,315	99.77	2
WP_035141397.1	Clostridium tetani	1,315	99.01	2
WP_039262284.1	Clostridium tetani	1,315	98.4	3
WP_115606337.1	Clostridium tetani	1,315	99.01	3
WP_129028853.1	Clostridium tetani	1,319	97.11	1
WP_129034268.1	Clostridium tetani	1,315	98.47	3
WP_129031034.1	Clostridium tetani	1,319	97.57	1
WP_129032368.1	Clostridium tetani	1,319	97.19	2
WP_129028331.1	Clostridium tetani	1,318	98.63	1
WP_129042638.1	Clostridium tetani	1,319	97.42	1
WP_130006254.1	Clostridium tetani	1,315	99.92	1

a Amino acid sequences from PubMed of the 13 extracted native toxin variants were analyzed. Assessment was conducted on 14 April 2020.

b aa, amino acids.

### 8MTT is nontoxic in mice.

**FIG 3:**
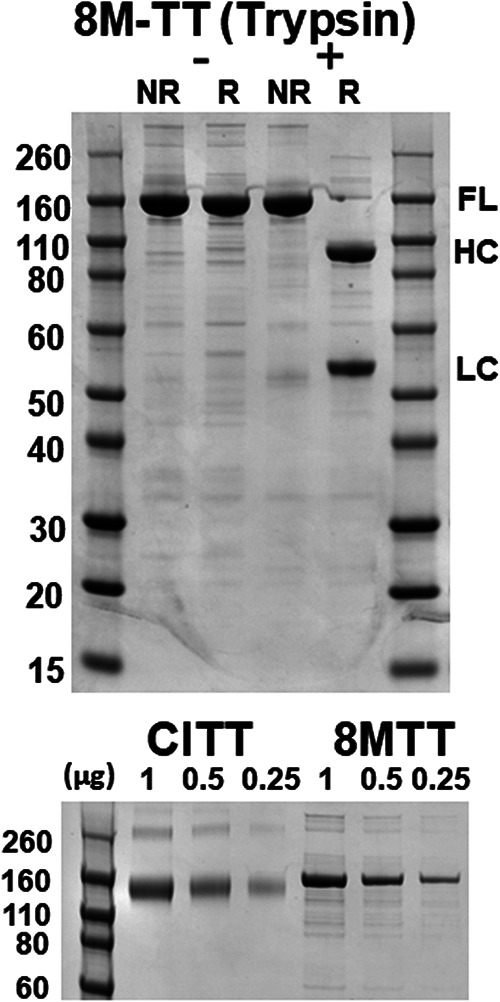
8MTT and CITT. (Top) 8MTT was expressed and purified from E. coli BL21(DE3). Three micrograms of purified 8MTT was incubated with trypsin (582231; Sigma) (1 μg to 100 μg ratio of trypsin to 8MTT) for 30 min at 35°C and subjected to SDS-PAGE alone (NR) or plus 0.1 M dithiothreitol (DTT) (R). FL, full-length 8MTT; HC, heavy chain; LC, light chain. (Bottom) The indicated amounts of chemically inactivated TT (CITT; Sigma) or 8MTT were subjected to SDS-PAGE. Both gels were fixed and stained with Coomassie blue, which are shown.

8MTT was expressed as a His_6_ protein in E. coli and purified by affinity chromatography and gel exclusion chromatography. Since posttranslational proteolytic cleavage of TT does not occur in E. coli, trypsin was used to convert single-chain 8MTT into a di-chain protein composed of a 50-kDa LC and a 100-kDa HC held together by an interchain disulfide bond (Fig. 3). Groups of four ICR outbred mice given an intraperitoneal (i.p.) injection of 0.6 mg per mouse of single-chain or trypsin-nicked di-chain 8MTT protein did not exhibit symptoms of tetanus intoxication and showed no signs of distress or pathology. Serial dilution of native TT showed that i.p. injection of six picograms represented 1× LD_50_ in ICR mice. Thus, 8MTT was calculated to be >50 million-fold less toxic than native tetanus toxin in this mouse model of tetanus.

### IgG response and protection from a native TT challenge following a single boost of 8MTT or CITT.

**FIG 4:**
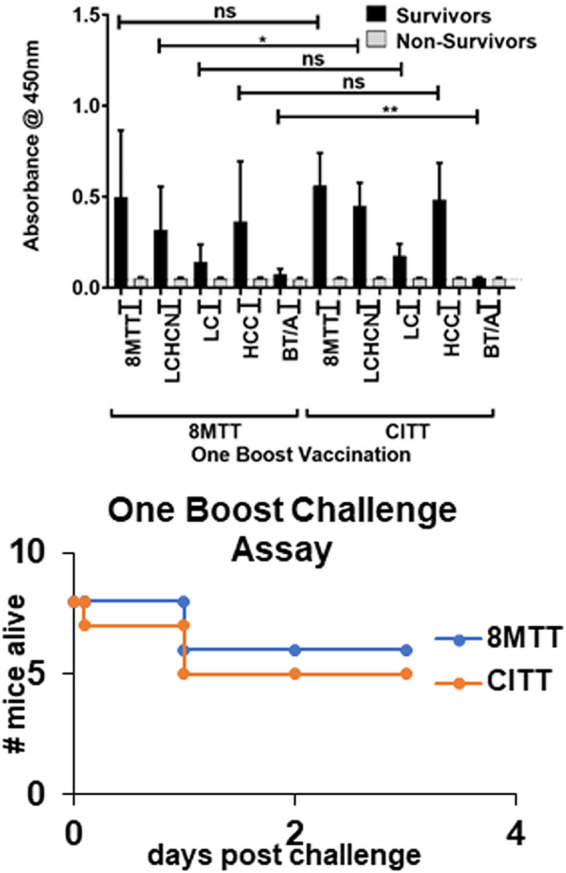
ELISAs of sera from mice surviving or not surviving native TT challenge following one boost of 8MTT or CITT. Mice (8 per group) were vaccinated subcutaneously with 0.1 μg of 8MTT or CITT in alum on days 1 and 28, on day 35 mice were bled, and on day 49 mice were challenged with 10,000× LD_50_ i.p. of native tetanus toxin. (Top) Sera (1/2,000 dilutions) from one-boost vaccinations were subjected to an ELISA using 250 ng of 8MTT, LCHCN/T (LCHCN), LC/T (LC), HCC/T (HCC), or 3MBT/A nontoxic botulinum toxin serotype A (BT/A) as antigens for 1 h at room temperature (RT). Plates were washed and incubated with goat anti-mouse IgG-horseradish peroxidase (1/20,000 dilution) at RT, washed, and incubated with Thermo Ultra-TMB substrate for 30 min, and the reactions were terminated with H_2_SO_4_. ELISAs were read according to *A*_450_, and data were analyzed for statistical significance, using GraphPad Prism. *, *P* < 0.05; **, *P* < 0.01. (Bottom) Times of survival for mice after a one-boost vaccination with 8MTT or CITT following challenge with 10,000× LD_50_ i.p. of native TT are shown.

Mice were vaccinated with equal primary doses of 8MTT or CITT (Fig. 3) (0.1 μg in alum) followed by one boost of 8MTT or CITT (0.1 μg in alum). Enzyme-linked immunosorbent assay (ELISA) assessment of IgG in blood collected at 7 days postboost showed that 8MTT and CITT elicited similar IgG responses to those of the antigens used in the ELISA, which included TT > light chain (LC) linked to the translocation domain (HCN) (LCHCN) = HCC > LC. Six of eight mice vaccinated with one boost of 8MTT and five of eight mice vaccinated with one boost of CITT survived challenge by 10,000× LD_50_ i.p. of native TT, with the time of survival longer for the 8MTT-vaccinated mice than for the CITT-vaccinated mice following native TT challenge (Fig. 4).

**FIG 5:**
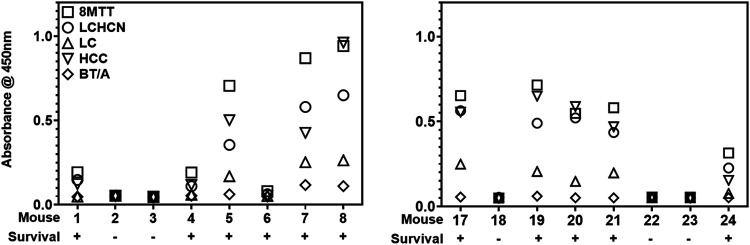
ELISAs of sera from individual mice surviving or not surviving native TT challenge following vaccination with one boost of 8MTT or CITT. Mice (8 per group) were vaccinated subcutaneously with 0.1 μg of 8MTT or CITT in alum on days 1 and 28, on day 35 mice were bled, and on day 49 mice were challenged with 10,000× LD_50_ i.p. of native tetanus toxin. Sera (1/2,000 dilutions) from one-boost vaccinations were subjected to an ELISA using 250 ng of 8MTT, LCHCN/T (LCHCN), LC/T (LC), HCC/T (HCC), or 3MBT/A (BT/A) as antigens for 1 h at RT. Plates were washed and incubated with goat anti-mouse IgG-horseradish peroxidase (1:20,000 dilution at RT), washed, and incubated with Thermo Ultra-TMB substrate for 30 min, and the reactions were terminated with H_2_SO_4_. ELISAs were read according to *A*_450_.

ELISA assessment of the IgG responses in individual mice showed that protection from TT challenge correlated with the production of an IgG response to either the 8MTT or CITT vaccination (Fig. 5), whereby the absolute magnitude of the IgG response did not differentiate survival from challenge by 10,000× LD_50_ i.p. of native TT. Relevant to the overall immunogenicity of TT for mice responding to 8MTT or CITT vaccinations, the IgG response to LC was consistently lower than the IgG response to either the LCHCN or HCC. This implies that the HCN and HCC are more immunogenic than LC. Also, the orders of the magnitude of the IgG responses to HCN or HCC varied among individual mice vaccinated with either 8MTT or CITT.

### IgG response and protection from a native TT challenge following two boosts of 8MTT or CITT.

In an independent experiment, outbred ICR mice were vaccinated with a primary dose of 8MTT or CITT (0.1 μg in alum) (Fig. 3) followed by two boosts of the 8MTT or CITT. Mice were bled 7 days after the first and second boosts and then challenged with 800,000× LD_50_ i.p. of native TT after the second boost.

**FIG 6:**
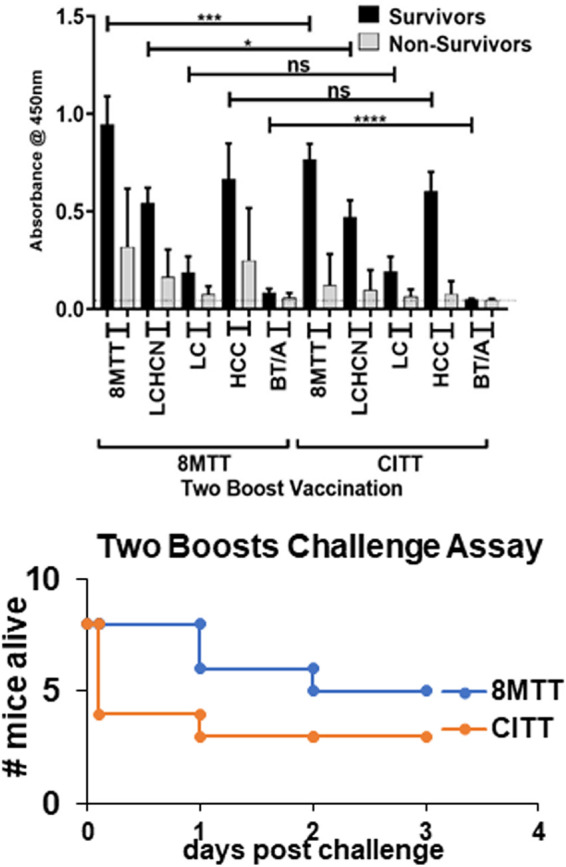
ELISAs of mice challenged with native TT after two boosts of 8MTT or CITT. Mice (8 per group) were vaccinated subcutaneously with 0.1 μg of 8MTT or CITT in alum on days 1, 28, and 56. On day 35 (first boost) and day 62 (second boost) mice were bled, and on day 69, mice were challenged with 800,000× LD_50_ i.p. of native tetanus toxin. (Top) ELISAs of sera from individual mice vaccinated with two boosts of 8MTT or CITT were performed against 8MTT, LCHCN/T (LCHCN), LC/T (LC), HCC/T (HCC), or 3MBT/A (BT/A) as described for Fig. 4. Averages of the *A*_450_ with standard deviations (SDs) are shown. (Bottom) Times of survival for mice after two-boost vaccinations with 8MTT or CITT following challenge with 800,000× LD_50_ i.p. of native TT are shown.

After the first boost, 8MTT and CITT elicited similar IgG titers to TT, which were >LCHCN = HCC > LC (Fig. 6). After the second boost, 8MTT elicited stronger IgG responses to TT, LCHCN, and HCC than CITT (Fig. 6 and Table 3). Five of eight mice vaccinated with two boosts of 8MTT and three of eight mice vaccinated with two boosts of CITT survived challenge by 800,000× LD_50_ i.p. of native TT (Fig. 6). ELISA showed that after two boosts, mice surviving the TT challenge had a stronger IgG response to TT than nonsurvivors, and the time of survival was longer for the 8MTT-vaccinated mice than for the CITT-vaccinated mice following native TT challenge (Fig. 6).

**TABLE 3:** Statistical significance in the IgG titers of mice boosted once or twice with 8MTT or CITT^*a*^

Domain analyzed	Mean diff. (*A*_540_)^*b*^	Significant?	Adjusted *P* value
8MTT vaccination			
TT vs TT (8MTT)	0.43	Yes	<0.0001
LCHCN vs LCHCN	0.24	Yes	<0.0001
LC vs LC	0.078	No	0.4647
HCC vs HCC	0.35	Yes	<0.0001
BT/A vs BT/A	0.028	No	0.9991
CITT vaccination			
TT vs TT (8MTT)	0.24	Yes	<0.0001
LCHCN vs LCHCN	0.12	Yes	0.0362
LC vs LC	0.05	No	0.907
HCC vs HCC	0.17	Yes	0.0006
BT/A vs BT/A	0.003	No	>0.9999

a As described for Fig. 6, mice were vaccinated subcutaneously with 0.1 μg of 8MTT or CITT in alum on days 1, 28, and 56 and bled at day 35 (first boost) and day 62 (second boost) followed by a challenge with 800,000× LD_50_ i.p. of native TT on day 69 and scored for survival. ELISAs were performed on sera from individual mice for IgGs against 8MTT, LCHCN/T (LCHCN), LC/T (LC), HCC/T (HCC), or 3MBT/A (BT/A). Statistics (ordinary one-way ANOVA [GraphPad Prism 8] on the relative IgG responses between the first and second boosts are shown.

b Difference in IgG titers between boost 1 and boost 2.

**FIG 7:**
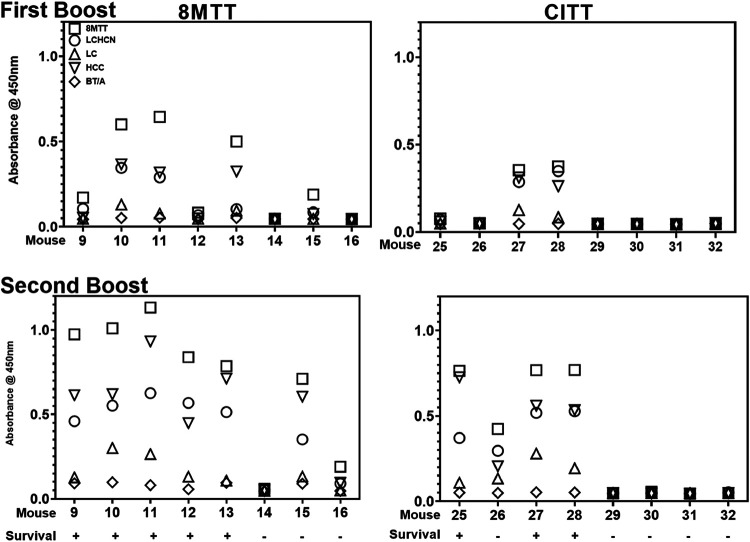
ELISAs of individual mice surviving or not surviving native TT challenge after two-boost vaccination with 8MTT or CITT. Mice (8 per group) were vaccinated subcutaneously with 0.1 μg of 8MTT or CITT in alum on days 1, 28, and 56 and bled at day 35 (first boost) and day 62 (second boost) followed by a challenge with 800,000× LD_50_ i.p. of native TT on day 69 and scored for survival. ELISAs were performed on sera from individual mice for IgG against 8MTT, LCHCN/T (LCHCN), LC/T (LC), HCC/T (HCC), or 3MBT/A (BT/A). Statistics for the IgG responses between the first and second boosts are shown in Table 3.

ELISA assessment of the IgG responses in individual mice showed that protection from TT challenge correlated with the production of an IgG response to either the 8MTT or CITT vaccination (Fig. 7), whereby the absolute magnitude of the IgG response differentiated survival from challenge by 800,000× LD_50_ i.p. of native tetanus toxin. Mice surviving the TT challenge after the second boost of 8MTT or CITT had the highest IgG responses within each individual group. Unlike the first boost and challenge, a detectable IgG response to TT was not sufficient to protect from the 800,000× LD_50_ TT i.p. challenge, suggesting a threshold immune response had to be established to survive this high-dose challenge. Also relevant were the findings that within each group of individual mice, there was a range of responders to 8MTT or CITT vaccination, where mice that mounted a detectable immune response after the first boost elicited a stronger IgG response to TT after the second boost than mice that failed to mount a detectable immune response to 8MTT or CITT following the first boost vaccination. Thus, within ICR outbred mice, there are strong responders and weak responders to 8MTT or CITT vaccination, with the variability being greater for the CITT-vaccinated mice than the 8MTT-vaccinated mice. This may be a quantitative effect of the vaccine, with 8MTT exposing more epitopes than CITT, or a qualitative effect, where 8MTT possesses similar epitopes that have been modified within the CITT vaccine through the process of formaldehyde inactivation.

## DISCUSSION

This study reports the engineering of a full-length genetically inactivated TT (8MTT) with functions for the three biological activities of TT, VAMP2 binding and cleavage, LC translocation, and dual host ganglioside receptor binding, inactivated by targeted single point mutations (Table 1). 8MTT was stable and purified as a single-chain 150-kDa protein that was nicked by trypsin into a disulfide-linked di-chain protein. 8MTT was not toxic in an outbred mouse model for tetanus. 8MTT was a potent vaccine against challenge by native TT and elicited a strong immune response relative to that from CITT. Assessment of individual mice also showed varied responses to 8MTT or CITT vaccination, with high IgG responders versus low IgG responders to vaccination. By the second boost in the two-boost experiment, seven of eight mice vaccinated with 8MTT had mounted an IgG response to TT, while four of eight vaccinated with CITT had mounted an immune response to TT. Thus, 8MTT is a potent vaccine against native TT challenge and could serve as a conjugate vaccine platform to enhance the immune response to polysaccharides and other macromolecular molecules (37–39) in a rapid response to a microbial pathogen.

Current CITT vaccines are clinically effective but are crude and contain varied amounts of CITT (7). Mass spectrometric analyses of five human CITT vaccines identified 991 *C. tetani* proteins, of which 206 proteins were common to the five vaccines (7). In addition, tetanus toxoid content varied, being 14% to 76% of the total *C. tetani* protein content among the individual vaccines (7). An analogous analysis of commercial diphtheria toxoids reported the presence of hundreds of Corynebacterium diphtheriae proteins as well as variable amounts of diphtheria toxoid in commercial diphtheria toxoid vaccines (40). In addition to content variability, the CDC reports common side effects of diphtheria toxoid-tetanus toxoid vaccination in adolescents and adults, including pain, redness, local swelling, fever, headache, and tiredness (41). Genetically inactivated TT and diphtheria toxin are anticipated to have improved purity along with a reduction of undesired symptoms due to undefined bacterial components contained in the vaccines.

The supply of tetanus vaccines depends on confirmation of CITT vaccine potency. WHO recommendations are used to confirm new batches of CITT vaccine potency (42). Since the first description of the flocculation unit as a tool to standardize a unit of CITT (43) and as more recently described (44), several approaches have been designed to modernize the flocculation unit. For example, Lyng and coworkers described the utility of single radial immunodiffusion and rocket immunoelectrophoresis (45) and simplified comparative assays in mice and in guinea pigs (46). In addition, consortiums have established approaches to replace the international standard for the tetanus toxoid (47). One of the advantages of utilizing a genetically engineered tetanus vaccine would be the ability to apply an extinction coefficient or nitrogen content to standardize the tetanus vaccine. This should expand the utilization of TT vaccine and TT conjugate vaccines. This will also be significant in the utilization of TT as a vaccine conjugate, as conjugation efficiency is highly dependent on purity and concentration of the protein component.

The presence of a genetic component toward the host response to foreign antigens has been documented in mice. Studies on pertussis reported a genetic component for the IgG subclass production to infection (48). A recent study of diphtheria–tetanus–acellular-pertussis (DTaP) vaccination in inbred and outbred mice reported IgG subclass responses to components of the vaccine differed according to titers and duration (49). This study reported differences among the strains tested for the potency of an immune response that was antigen specific and others that were antigen independent. Thus, the observed variation between the host response to 8MTT and CITT supports a genetic component to the host response to tetanus vaccination.

While 8MTT vaccination stimulated more outbred mice to mount a detectable immune response to TT than CITT vaccination, the detected immune response was not universal, where one mouse from the second boost of 8MTT and four mice from the second-boost CITT vaccination failed to elicit detectable IgG responses to tetanus toxin. Several studies have used serological reactivity to predict the state of immune protection provided by the diphtheria toxoid and tetanus toxoid vaccination, implying that humans also exhibit varied immune responses to CITT vaccination. In a measurement of serology by the Third National Health and Nutrition Examination Survey (1988 to 1994) of a cross-sectional sample of the U.S. population that included 18,045 people aged 6 years or older (50), 60.5% of the population had protective levels of diphtheria antibody and 72.3% had protective levels of tetanus antibody (>0.15 IU/ml) (51) according to WHO standards. In a breakdown of age groups, 91% of the U.S. population aged 6 to 11 years had protective levels of both diphtheria and tetanus antibodies. One interpretation of these outcome data is that ∼9% of the U.S. population may be low responders of CITT vaccination. Using a less stringent definition of immune protection, <0.01 IU/ml, another outcomes study reported that based upon antibody titers in a population of 540 subjects of known ages, there was 97% protective immunity to tetanus intoxication (52) with a 14-year half-life of titer. The 3% of the U.S. population that failed to establish protective immunity to tetanus intoxication represents a significant number of individuals susceptible to TT intoxication. While further studies are needed to evaluate percent population protection potential by 8MTT versus CITT, the data presented in this small pilot study indicate that 8MTT protected a greater percentage of mice than CITT.

In the present study, 8MTT is described as a potent prototype of a genetically engineered vaccine that is a model for the development of other bacterial toxin vaccines by using known structure-function properties of the toxins, such as diphtheria toxin (53) or pertussis toxin (54), as genetically engineered vaccines. These genetically engineered vaccines will be safe to produce pure and potent vaccine as well as candidate conjugate vaccine carriers.

## MATERIALS AND METHODS

### Production of recombinant proteins.

DNA that was optimized for E. coli expression, including 8MTT, light chain-translocation domain of tetanus toxin (LCHCN/T), light chain of tetanus toxin (LC/T), receptor-binding domain of tetanus toxin (HCC/T), and nontoxic botulinum toxin serotype A (BT/A) (30), and a genetically inactivated full-length botulinum toxin serotype A variant, was expressed in pET28 with a His tag at the N terminus in BL21(DE3) cells. Cells were grown in culture at 37° for ∼3 h at 250 rpm to an optical density at 600 nm (OD_600_) of ∼0.6 and then induced with 1 mM isopropyl-β-d-thiogalactopyranoside (IPTG) followed by overnight shaking at 20°C and 250 rpm. Cells were harvested in 20 mM Tris-HCl (pH 7.9), 0.5 M NaCl, and 5 mM imidazole. Cells were lysed with a French press and clarified by centrifugation and 0.45 μM cellulose acetate filtration (Thermo Fisher). Lysates were purified using Ni^2+^-nitrilotriacetic acid (NTA) resin equilibrated in lysis buffer. The column was washed with increasing imidazole concentrations (5 mM up to 20 mM) in 20 mM Tris-HCl (pH 7.9), 0.5 M NaCl. The NTA eluent was purified on a S300HR (S200HR for LC/T and HCC/T) size-exclusion Sephacryl column (550-ml column; Sigma) equilibrated in 10 mM Tris-HCl (pH 7.9), 0.2 M NaCl. Peak fractions were pooled and applied to a fresh Ni^2+^-NTA resin column (Qiagen) equilibrated in 10 mM Tris-HCl (pH 7.9), 0.2 M NaCl. Eluent was then dialyzed into 10 mM Tris-HCl (pH 7.9), 0.2 M NaCl, and 40% (vol/vol) glycerol and stored at −20°C. 8MTT purified by Ni-chromatography followed by gel filtration chromatology limited lipopolysaccharide (LPS) contamination. Purified 8MTT (100 ng) contained <0.2 EU of LPS (88282; Pierce), which is below the amount of LPS that elicits adjuvant activity (55, 56).

### Production of TT for mouse challenge.

*C. tetani* strain 64001 Lanzhou from the Eric A. Johnson culture collection was used to inoculate Hungate tubes containing 10 ml of modified Latham medium (57, 58) with 0.5 g Difco cooked meat medium. Tubes were incubated 18 h at 35°C, and 2 ml of the actively growing culture was used to inoculate 1.5 liters of modified Latham medium. Native TT was purified as described by Ozutsumi (59). After incubation for 42 h at 35°C, cells from the 1.5 liters of *C. tetani* culture were collected by centrifugation at 5,000 × *g* for 10 min at 4°C. Cell pellets were washed twice with 150 ml cold 0.15 M NaCl, and the cells were again collected by centrifugation at 5,000 × *g* for 10 min. TT was extracted from the cells by gently stirring the washed cell pellet overnight at 4°C in 100 ml 0.1 M sodium citrate (pH 7.5) containing 1.0 M NaCl. The overnight extract was centrifuged at 8,000 × *g* for 15 min at 4°C, and the supernatant containing single-chain TT was made 40% saturated with ammonium sulfate. To convert the single-chain toxin to the fully active di-chain form, the ammonium sulfate-precipitated extract was centrifuged at 8,000 × *g* for 30 min at 4°C, and the pellet was solubilized in 12 ml of 25 mM sodium phosphate buffer (pH 7.4) containing 0.1 M NaCl. Trypsin (TRTPCK; Worthington) was added at a concentration of 1 μg per ml of solubilized extract and incubated for 30 min at 35°C. Soybean trypsin inhibitor (Millipore Sigma) was added at 4 μg per ml to stop the digestion. The conversion of single-chain TT to the di-chain form was confirmed by Western blotting (data not shown). Specific toxicity (LD_50_ per milliliter) was determined by intraperitoneal injections of mice with serial dilutions of the trypsin-treated extract.

### Vaccine challenge.

Female ICR mice (18 to 22 g) were immunized subcutaneously with either 0.1 μg 8MTT or CITT (582231; Sigma) (16 mice per group), mixed with a 0.1× volume of alhydrogel as an adjuvant, on days 1 and 28. Eight mice per group were challenged with 10,000× 50% lethal doses (LD_50_) native TT on day 49. The other eight mice per group were given a second boost at day 56 with 0.1 μg 8MTT or CITT and challenged 13 days later with 800,000× LD_50_ i.p. Sera were collected 7 days prior to challenge. All animal experiments were approved by the Animal Care and Use Committee at the University of Wisconsin—Madison.

### ELISA.

8MTT, LCHCN/T, LC/T, HCC/T, BT/A, a genetically engineered or nontoxic full-length botulinum toxin (30) (250 ng per well) or no protein was added to 100 μl of coating buffer (50 mM Na_2_CO_3_ [pH 9.6]) to enzyme-linked immunosorbent assay (ELISA) plates (number 9018; Corning) and incubated overnight at 4°C. Plates were washed three times with 300 μl of phosphate-buffered saline (PBS) and blocked for 30 min at room temperature with 200 μl of PBS plus 1% (wt/vol) bovine serum albumin (BSA) per well. Plates were incubated with mouse antisera diluted 1/2,000 (100 μl) from mice immunized with 8MTT or CITT for 1 h at room temperature in PBS plus 1% BSA. After washing three times with 300 μl of PBS, plates were incubated for 1 h at room temperature with goat anti-mouse IgG-horseradish peroxidase (IgG-HRP) (1:20,000; Thermo) in PBS and 1% BSA. Plates were washed three times with 300 μl of PBS, and wells were incubated with 100 μl of tetramethyl benzidine (TMB; Thermo Ultra TMB) as the substrate. Reactions were terminated after 30 min with 100 μl of 0.1 M H_2_SO_4_, and absorbance was read at 450 nm with a Victor3 V plate reader (Perkin Elmer).

### Statistical analysis.

Data were analyzed for statistical significance, using a *t* test for grouped sera and an ordinary one-way analysis of variance (ANOVA) assuming normal distribution for 1- versus 2-boost experiments, using GraphPad Prism 8.
